# Visualizing faculty development impact: A social network analysis

**DOI:** 10.1007/s40037-019-0510-9

**Published:** 2019-04-18

**Authors:** Yang Yann Foo, James Moody, Sandy Cook

**Affiliations:** 1 Duke-NUS Medical School, Academic Medicine Education Institute, Singapore, Singapore; 2 Department of Sociology, Duke University, Durham, USA; 3 King Abdulaziz University, Jeddah, Saudi Arabia

**Keywords:** Faculty development, Program evaluation, Social network analysis

## Abstract

Faculty development programs have tended to focus on low levels of evaluation such as participant satisfaction rather than assess the actual changes that training has brought about in the workplace. This has prompted scholars to suggest using social network analysis as a means to provide a more rigorous method of evaluating the impact of faculty development. To test the feasibility of such a suggestion, we used the social network analysis concepts of social cohesion to assess the impact of a year-long fellowship program conducted by Duke-NUS Medical School’s Academic Medicine Education Institute (AM·EI). Specifically, we used the key metrics of connectedness and betweenness centrality to assess the changes in the AM·EI fellows’ information and collaboration networks post-fellowship. We invited three cohorts of AM·EI fellows (2013–2016; *n* = 74) to participate in a branched survey. The response rate was 64%; *n* = 47. Results showed that in terms of connectedness, the largest connected set more than doubled in size, and pair level reachability grew threefold. Betweenness centrality among the AM·EI fellows also increased, with more individuals reporting that they sought advice from the fellows as well as trusted the advice the fellows provided. In sum, this study suggests that it is indeed viable to use social network analysis to identify changes in social cohesion. As such, social network analysis serves as another tool for scholars to use to assess the impact of their faculty development efforts.

## Background

Faculty development programs, though costly and time-consuming, are implemented in most organizations because training is deemed important for organizational growth [1]. Although the evaluation of the impact of faculty development is increasingly mandated, it remains elusive [2] and underexplored [3] because many studies in this area have thus far focused on participant satisfaction with faculty development programs rather than observations of change [4]. To address this gap, faculty development scholars have suggested using social network analysis as a more rigorous method to unpack the complexity of the roles of faculty development participants in fostering change in the workplace [5].

It makes sense to propose using social network analysis as an evaluation method as it provides a framework with which to examine how an individual’s behaviour feeds into a larger web of social connections [6]. In the field of medical education, social network analysis has been shown to be a viable tool with which to unveil the invisible impact of informal social interaction on student learning [7]. As applied to faculty development evaluation, networks would provide a way to identify cohesiveness among individuals within a network that can then be linked to performance.

Our study is a proof-of-concept paper aiming to show the feasibility of using social network analysis as a method to evaluate faculty development impact.

## Innovation methods

We used social network analysis concepts of social cohesion, specifically the key metrics of connectedness and betweenness centrality, to examine the impact of a year-long fellowship program conducted by Duke-NUS Medical School’s Academic Medicine Education Institute (AM·EI). Social network analysis uses nodes to denote individuals [8]. Connectedness measures the number of ties the nodes have in a network, while betweenness centrality measures the number of times a node acts as a link along the shortest path between two other nodes in a network [8]. We chose to focus on a longitudinal fellowship as such programs have been shown to be more effective at helping participants build trust and relationships, which in turn motivated and enthused participants to collaborate—post-program—in the workplace [9].

Our hypothesis was that, post-fellowship, collaboration among the AM·EI fellows, their colleagues and associates at work would increase by means of their advice-seeking and advice-giving behaviour regarding teaching and learning activities. These changes would be indicated in social network analysis graphs where nodes representing AM·EI fellows would change in terms of connectedness and betweenness centrality.

## Survey instrument

We built a branched survey based on social network analysis literature looking at information and collaboration networks in the workplace [10–12]. We asked questions about from whom and to whom our AM·EI fellows sought and gave advice related to teaching and learning matters before and after the AM·EI fellowship. We also included questions which asked why the AM·EI fellows chose to collaborate with the named individuals. A sample of the questionnaire can be found at https://mysurvey.nus.edu.sg/EFM/se/548BF73609D80E2C.

To validate our survey instrument, we conducted cognitive interviews [13] with 10 AM·EI fellows whom we recruited on a voluntary basis. This was to ensure that the participants would find it easy to understand the phrasing used in the survey questions, and that the questions were relevant to their AM·EI fellowship experience.

The cognitive interviews were conducted in three phases over 3 weeks. In week 1, four fellows were interviewed about their experience completing the survey online. Based on their feedback, we modified the survey questions. In week 2, we asked another three fellows to complete the modified survey and interviewed them on their user experience. In week 3, we repeated the process and stopped refining the survey when there were no further suggestions. To ensure that we captured the fellows’ individual user experience, all the interviews were conducted separately. To prevent important feedback from being forgotten, we interviewed each of them immediately after survey completion.

Through cognitive interviews, we were able to refine the clarity of our questions. For instance, our original phrasing for question 4 was this: ‘Before joining the AM•EI fellowship, to whom have you given advice related to education matters?’ In week 1, two of the first group of four fellows gave answers that included groups of unnamed people, such as their residents. As our study sought to examine the change in social cohesion among individuals, we sought to offer greater clarity in our survey question by adding in parenthesis the word ‘(individual)’ to our question. In week 2, two of the AM·EI fellows found the phrase ‘education matters’ unclear. To clarify, we changed ‘education matters’ to ‘teaching and learning matters’, which was more specific.

Without refining our survey, we would probably have found that a significant portion of the data we collected was unusable. Cognitive interviews helped us to forestall that problem.

## Program and participants

Developed by AM·EI faculty members, the fellowship focused on refining the educational acumen of clinical educators with significant teaching responsibilities. Over 1 year from October to the following September, the AM·EI fellows met 10 times, once a month. No sessions were run in June and December because these were school holiday months in Singapore.

For our study, we invited 74 AM·EI fellows from three of our cohorts to take part in our survey (Cohort 1 *n* = 28; Cohort 2 *n* = 22; Cohort 3 *n* = 24). The AM·EI fellows came from SingHealth—a healthcare group in Singapore—and comprised physicians, nurses, and allied health professionals. All of them have a significant teaching portfolio in their respective departments.

The content of the AM·EI fellowship was informed by the Academy of Medical Educators’ professional standards (http://www.medicaleducators.org/Professional-Standards) and focused on the following areas: (1) designing and planning, (2) teaching and facilitating learning, (3) assessing learning, (4) educational research and scholarship, and (5) educational management and leadership. We used the learning strategy of flipped classroom and as such gave pre-readings to the AM·EI fellows. They worked on group projects and faculty members also observed their teaching sessions for peer evaluation purposes.

## Data collection and analysis

Between March and May 2017, we emailed the survey to our three cohorts of AM·EI fellows. Those who did not respond were sent no more than two reminders to participate in the survey. As incentives, we offered the AM·EI fellows vouchers of SGD10 dollars. These were issued by an administrator upon completion of the survey.

We analyzed the data using SAS version 9.4 (Copyright (c) 2002–2012 by SAS Institute Inc., Cary, NC, USA.) where we constructed scores by applying standard social network cohesion and connectivity metrics to the adjacency matrix [8]. The figures were done using Pajek and Illustrator.

## Ethics

In the email sent to the AM·EI fellows, we indicated the voluntary nature of their participation in this study. In addition, we prefaced our survey with informed consent information. Survey respondents who decided not to take part after reading the informed consent information were brought to a termination page where they exited the survey.

We sought ethics clearance from SingHealth’s Centralized Institutional Review Board on 5 August 2016, reference number—2016/2656 and approval was waived.

## Evaluation

### AM·EI fellows’ connectedness post-fellowship

**Fig. 1:**
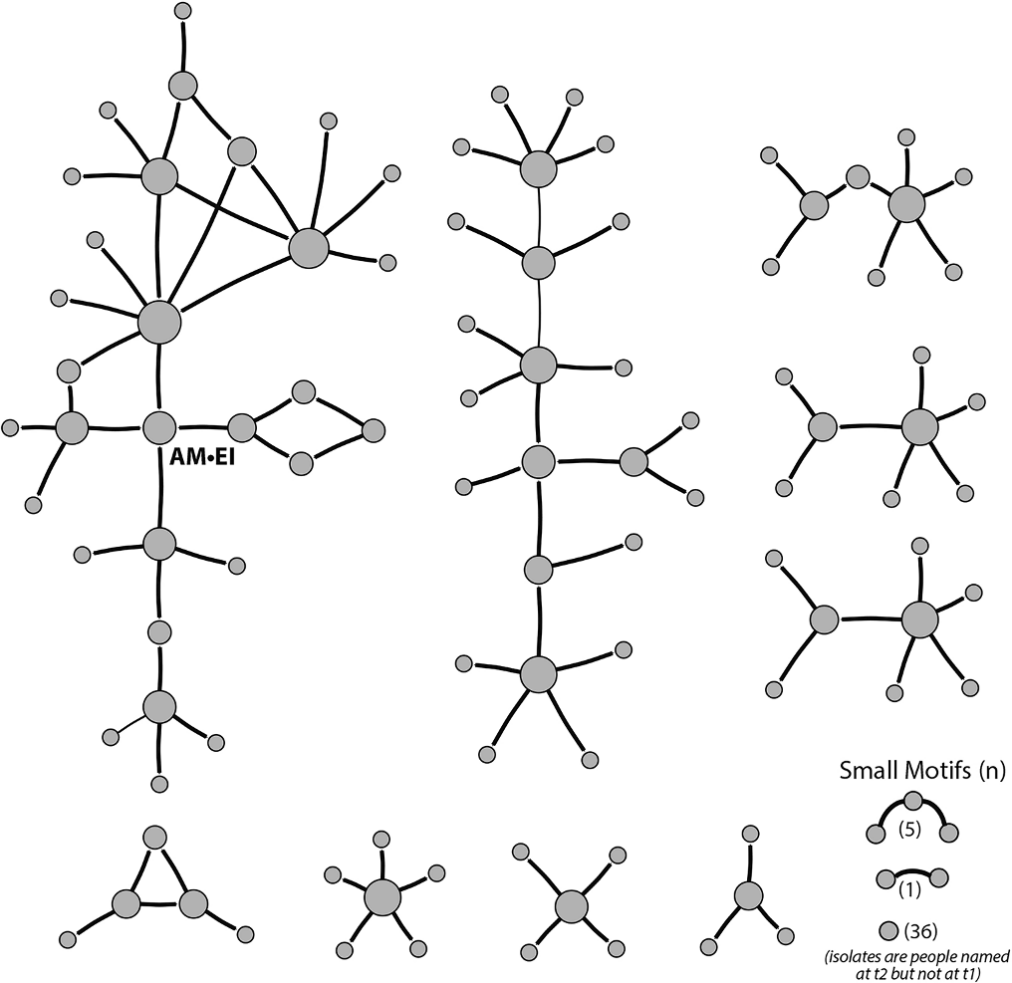
Time 1 (T1) AM·EI fellows’ network *before* fellowship

**Fig. 2:**
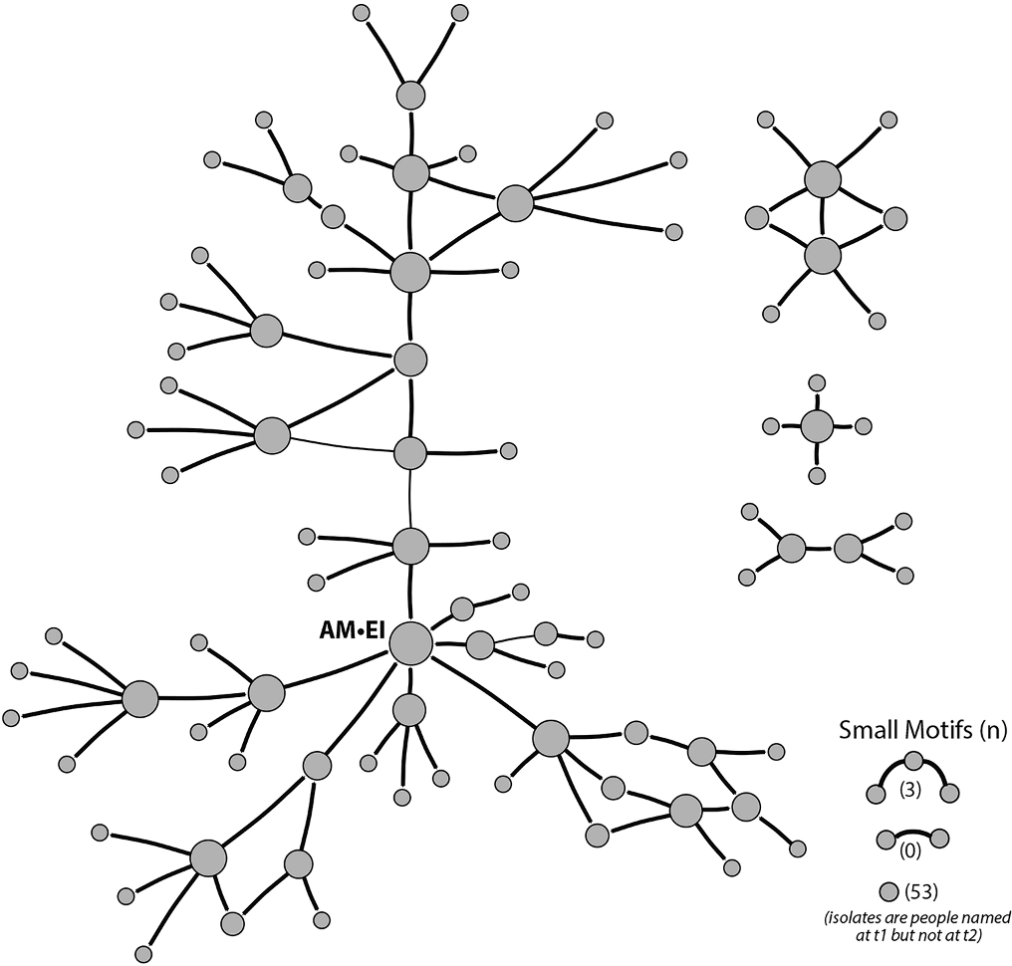
Time 2 (T2) AM·EI fellows’ network *after* fellowship

Of a total of 74 AM·EI fellows, 47 (64%) completed our survey. Figs. 1 and 2 represent the social network graphs before and after the AM·EI fellows completed the fellowship. The nodes are not named for de-identification purposes. They represent AM·EI fellows, and the individuals the fellows named in their surveys from and to whom they sought and gave advice on teaching and learning matters. The only exception is the node named AM·EI: it represents all the faculty members involved in teaching in the AM·EI fellowship. Tab. 1 and 2, respectively, present the cohesion statistics for AM·EI fellows, and the frequency with which AM·EI fellows sought and provided educational advice regarding teaching and learning activities, post-fellowship.

**Table 1:** Cohesion statistics for AM·EI fellows post-fellowship

	Definition	Time 1, Persons	Time 2, Persons
Nodes	Number of nodes in the network	152	152
Ties per respondent	Average number of nominations per survey respondent (*n* = 47)	2.34	2.17
Largest component	Largest set of people who could reach each other in the network	30 (30/152 = 19.7%)	71 (71/152 = 46.7%)
Reachability	Proportion of pairs that can reach each other in the network	7%	22%

**Table 2:** Frequency AM·EI fellows sought and provided educational advice

	Advice sought			Advice given		
	Time 1	Time 2	*P*-value	Time 1	Time 2	*P*-value
They/I had the information I/they needed	2.98	3.42	0.05	2.97	3.25	0.16
They/I responded to my/their request for help	2.78	3.00	0.14	2.66	3.33	0.06
I/They trust them/me	2.79	2.94	0.40	2.96	3.17	0.01

Overall, the volume of social relations over time remained essentially unchanged (Tab. 1: Time 1, Persons—2.34 nominations per respondent; Time 2, Persons—2.17) nominations per respondent. However, the social organization of relations changed quite dramatically as can been seen in Figs. 1 and 2, which represent the social network graphs before and after the AM·EI fellows completed the fellowship. In particular, relations in T1 (Fig. 1) were more fragmented, with the largest connected set containing only 30 out of 152 persons (Tab. 1: 19.7%) and only 7% of pairs could reach each other through the networks. By contrast, after the fellowship, the size of the largest component more than doubled to 71 nodes (Tab. 1: 46.7% of nodes) and pair level reachability increased by over threefold to 22.%.

## Betweenness centrality and frequency AM·EI fellows sought or provided advice

After the fellowship, AM·EI’s ability to link otherwise disconnected nodes increased, with its betweenness centrality increasing from 0.02 in Time 1 (Fig. 1) to 0.16 in Time 2 (Fig. 2). In terms of the frequency of advice sought and given, more individuals trusted the advice provided by the AM·EI fellows (Tab. 2: T1 = 2.96, T2 = 3.17, *p* = 0.01). Reported advice-seeking behaviour of the AM·EI fellows also increased (Tab. 2: T1 = 2.98, T2 = 3.42, *p* = 0.05). There was a slight but insignificant increase in the amount of advice the AM·EI fellows provided others in the network (Tab. 2: T1 = 2.66, T2 = 3.33, *p* = 0.06), and in the AM·EI fellows’ confidence about having the information others needed (Tab. 2: T1 = 2.97, T2 = 3.25, *p* = 0.16).

These results indicate that at the individual level, there was greater trust and participants were more likely to exchange information as needed. Also, since the program’s social structure is more connected, such information exchange likely spreads more easily through the community.

## Limitations

Our study has several limitations. The first is the retrospective nature of our survey. As we conducted our survey only 6 months to 2.5 years after the completion of the AM·EI fellowship, the time gap, especially for the first cohort, could have compromised the ability of some of our respondents to recall all the colleagues or associates with whom they had collaborated post-fellowship. Thus, the recall bias inherent in our retrospective survey has limited our ability to analyze fully the impact of the AM·EI fellowship.

The second limitation was our inability to provide a dropdown list of all the names of SingHealth staff in our online survey. Such a feature could arguably have enhanced the AM·EI fellows’ ability to recall the individuals with whom they shared information and collaborated. However, creating such a feature is challenging for our study which involves SingHealth, a Singapore healthcare group comprising four public hospitals, five national specialty centres, and nine polyclinics. Creating and using a dropdown list consisting of tens of thousands of names of SingHealth staff would be very onerous.

The third limitation concerns the non-respondents. Although our study’s 64% response rate is generally acceptable for surveys, and social network analysis scholars have also found that data missing at random [14] or systematically [15] will not lead to appreciable bias in cohesion indicators, the fact is that we do not know the impact of the fellowship on the remaining 36% of fellows. In short, we are unable to find out how the missing information from the 27 fellows would change the pattern of our data. Presumably some did not take part in the study because they had a negative experience participating in the fellowship, and this might be reflected in the social ties they have with other Fellows, colleagues and associates at work. Their connectedness to these individuals might have remained the same, or perhaps even diminished as compared with before.

Even though our study has limitations, we have nonetheless achieved our goal of demonstrating the feasibility of using social network analysis as a method to evaluate faculty development impact. However, future projects should be planned prospectively to circumvent the limitations discussed.

## Reflection

Our study has demonstrated that it is indeed viable to use social network analysis to evaluate the impact of faculty development programs in a rigorous manner. Specifically, we were able to identify how individuals’ ability to act as conduits to connect others in the network has changed and, secondly, track previously undetectable outcomes in terms of participants’ trust and cooperation levels with one another post-faculty development.

However, to limit recall bias, future faculty development studies using social network analysis should be planned prospectively, and the scope of evaluation should be restricted to specific departments or institutions so that surveys used to collect data could include the names of specific individuals.

In sum, compared with satisfaction surveys, social network analysis can serve as a rigorous alternative in the study of faculty development impact. Hopefully, this paper will give impetus to future research to move into a new phase where studies will examine the concrete benefits of training by identifying cohesiveness among individuals within a network that can then be linked to faculty development outcomes.
